# Microrefugia: Not for everyone

**DOI:** 10.1007/s13280-014-0599-3

**Published:** 2015-01-09

**Authors:** Kristoffer Hylander, Johan Ehrlén, Miska Luoto, Eric Meineri

**Affiliations:** 1 Department of Ecology, Environment and Plant Sciences, Stockholm University, 106 91 Stockholm, Sweden; 2 Department of Geosciences and Geography, University of Helsinki, 00014 Helsinki, Finland

**Keywords:** Climate-forcing factor, Limiting factor, Population, Refugia, Resilience, Topography

## Abstract

Microrefugia are sites that support populations of species when their ranges contract during unfavorable climate episodes. Here, we review and discuss two aspects relevant for microrefugia. First, distributions of different species are influenced by different climatic variables. Second, climatic variables differ in the degree of local decoupling from the regional climate. Based on this, we suggest that only species limited by climatic conditions decoupled from the regional climate can benefit from microrefugia. We argue that this restriction has received little attention in spite of its importance for microrefugia as a mechanism for species resilience (the survival of unfavorable episodes and subsequent range expansion). Presence of microrefugia will depend on both the responses of individual species to local climatic variation and how climate-forcing factors shape the correlation between local and regional climate across space and time.

## Introduction

During periods of climate change, many species ranges shift, contract, or expand due to changed temperature, moisture, or other climatic factors (Williams et al. 2004). Also the recent warming is reflected in changed distributions (Chen et al. 2011), especially for mobile species at their coldward (i.e., toward the cold end of a temperature gradient) altitudinal or latitudinal edges (Parmesan et al. 1999; Hickling et al. 2006; Bergamini et al. 2009; Thomas 2010; Elmhagen et al. 2015). After the last glacial maximum, many species recolonized formerly occupied areas in a pace much faster than predicted from dispersal models (Clark et al. 1998). Long-distance dispersal has been emphasized as an important mechanism explaining this phenomena (Hampe 2011). Recently, it has also been proposed that many species in fact survived in small pockets at some distance from their core refugial areas, so-called microrefugia, from where they recolonized surrounding areas once climatic conditions improved (Stewart and Lister 2001; Ashcroft 2010; Hampe and Jump 2011). Some species might even have survived only in such small, scattered microrefugia. Since microrefugia constitute a potential mechanism for increased resilience of species to climate change, a refined knowledge of where these sites are likely to be situated in a future climate and which species are likely to benefit from this mechanism is much needed (Dobrowski 2011; Corlett and Westcott 2013; Woolbright et al. 2014).

**Fig. 1:**
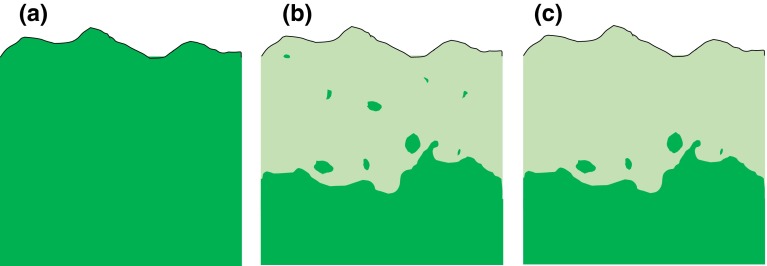
Species distribution before and after range contraction; **a** a species with a continuous distribution all the way until a barrier (in the north in this case). **b**, **c** After a climatic deterioration (from north in this case) the species retract southward. However, the species linger on in a number of microrefugia (smaller green areas north of the main distribution area). **b** Many microrefugia even far away, **c** few microrefugia close to main distribution. When only looking at the current distribution (e.g., **b** and **c ** panel) without knowledge of the past it is likely (but not necessary so) that the small isolated patches are remnants from a past wider distribution. However, it is still likely that the conditions in those places have similarities to conditions in the core distribution area

In this paper, we build on the concept by Rull (2009), who defined microrefugia as small areas outside the core distribution area where species persist despite the surroundings being inhospitable (Fig. 1). However, microrefugia may exist also in the absence of a core area if the distribution has contracted to only those small areas (see also Keppel et al. 2012). Here, we discuss scenarios where both a core distribution and microrefugia are present to illustrate how contrasting conditions across space can inform us about microrefugia also in a temporal context. Still, the points we make using this framework will be valid for many other circumstances, including “holdouts” defined as places in which species may persist for a long time after that conditions have deteriorated but where they eventually will go extinct (in contrast to microrefugia where they will survive and expand from) (Hannah et al. 2014).

For populations in microrefugia to play a role in subsequent range expansions, they need to persist until conditions outside microrefugia allow establishment (cf. Auffret et al. 2015). Individuals thus need to have a mean population growth rate of at least one so that they do not go extinct before conditions improve. Different mechanisms such as clonal growth, having a seed bank or seedling recruitment could all be important mechanisms for population persistence (Hylander and Ehrlen 2013). Studying the relationships between population growth rates and abiotic/biotic environmental variables across species’ distributions is therefore key to understand the conditions determining species distributions and abundances, and thus to identify potential microrefugia.

Several studies have highlighted the potential of a rough topography to increase the likelihood of species survival in microrefugia during unfavorable climatic episodes (Luoto and Heikkinen 2008; Loarie et al. 2009; Randin et al. 2009; Keppel et al. 2012). The logic is that in topographic heterogeneous landscapes there can be a large variation in local climate along various physiographic gradients, even down to the scale of meters (Scherrer and Körner 2011). The simplest example of how a species might survive despite a changed regional climate is when it can move along altitudinal gradients to track suitable conditions. Several studies have found upward shifts in the optimum or upper range limit for species in mountainous environments in response to recent warming (Lenoir et al. 2008; Bergamini et al. 2009; Felde et al. 2012). In a topographically heterogeneous landscape, species might therefore find places that possess the necessary climatic conditions after climate change, even if all areas change analogously (cf. Scherrer and Körner 2011). However, it is also likely that a rough topography results in not all areas changing equally, and that areas in which the change of the local climate (at scale of 1–100 m) is partly decoupled from the regional climate change (from the kilometer scale) might be common (Dobrowski 2011). Both of these mechanisms can thus be important not only during altitudinal, but also for latitudinal, range shifts in that species might be able to linger on in small places still remaining suitable at some distance from the new core range (Jump et al. 2009; Hampe and Jump 2011).

In this review, we propose that climatic variables generally influence species’ distributions and occurrences in microrefugia. An additional mechanism, not considered here, is that meta-population dynamics can restrict range limits so that suitable isolated habitats fragments are not occupied (Holt and Keitt 2000). It is also important to take into account that, for example, a patchy distribution of a certain type of bedrock can create an irregular distribution of microrefugia that has little to do with climatic variation (Keppel et al. 2012).

Here we review the prerequisites for microrefugia to play a role for species persistence during periods of generally unfavorable conditions along two main tracks: species distributions–climate relationships and physiography–climate relationships. We argue that simultaneous examinations of these two types of relationships are essential to judge the potential importance of microrefugia for species persistence, and that these issues have received limited attention although being potentially important (e.g., Dobrowski 2011; Hannah et al. 2014).

## How do climatic variables limit the distribution of species?

Examining current distributions of species can inform us about the potential for species to survive in microrefugia in spite of regional climatic changes. Not least, studies of processes shaping range edges are relevant to the topic of microrefugia. If we know more about how climate affects both the physiology and the ecology of species, especially at range edges, we are better equipped to predict future changes in their distribution.

According to Hutchinson’s definition, the niches of coexisting species cannot be identical (Hutchinson 1957). This implicitly suggests that two co-existing species should either respond to at least one different variable or to some extent respond differently to the same variables. Following on that argument, we would not expect to find general patterns in how species are limited by climate or utilize microrefugia. However, due to general constraints in metabolic and enzymatic processes, it might still be true that certain mechanisms for how climate limit species can be more common than others, particularly among related species. Still, most suggestions of generalizations regarding the factors determining range edges have numerous exceptions and the limiting factors seem to be largely species specific (Gaston 2009).

Climate limitation of species is evident from historical data on pollen and macrofossils which tell a coherent story of large distributional shifts in response to climatic shifts (Williams et al. 2004). Yet, it is not straightforward to identify the mechanism driving these patterns. One approach is to examine correlations between isotherms of different climatic variables and distribution limits (Grace 1987). To what extent such associations reflect causal relationships is however less clear. This is because a large number of more or less correlated candidate variables might lead to overfitting in statistical models (Gaston 2003).

Many studies have investigated specific aspects of climatic effects on single vital rates, such as effects of climate extremes on survival and of moisture and temperature on recruitment (see below). Yet, few studies have examined effects of climatic variation across space or time on estimates of the overall performance, e.g., in terms of the population growth rate (but see Doak and Morris 2010; Nicole et al. 2011; Salguero-Gomez et al. 2012). The ability of a species to survive under a given set of environmental/climatic conditions is determined by the population growth rate. A positive population growth rate (assuming no strong Allee-effects and no strong effects of stochasticity) determines the conditions under which the species can potentially survive. Because population growth rate is the integrated result of all vital rates, effects of climatic variables on distributions cannot be inferred from relationships between climate and single vital rates. A single climate factor could for example have opposite effects on different vital rates (demographic compensation), as shown for an alpine plant along a latitudinal gradient (Doak and Morris 2010). Causal relationships may often be difficult to infer from observational data and relationships between environmental factors and vital rates should therefore ideally be confirmed by manipulative experiments. To what extent an effect on a single vital rate could drive major distribution limitations is therefore still unclear, although many such propositions abound (see below). Notwithstanding the need to assess the effects of climatic variables on overall performance, studies on components of fitness might still be useful to understand the mechanisms underlying climate effects on species performance (e.g., Pigott and Huntley 1981).

There is a long history of studies on physiological constraints to species performance, and thus implicitly to their distributions (see e.g., Grace 1987; Turnock and Fields 2005). A common theme is if and how the coldward distributions of species can be limited by cold temperatures, especially during the winter (Grace 1987; Turnock and Fields 2005; Kollas et al. 2014). For example, it has been proposed that areas with regular winter temperatures of −15 °C could not harbor evergreen trees and shrubs (Woodward 1987). A common mortality cause for coniferous trees at high altitudes and latitudes is winter/spring drought because the trees cannot access water when the ground is frozen and the needles start to photosynthesize (Kullman 2007). However, mortality does not need to always be sudden. For example, if a plant has a negative carbon balance, it will eventually lead to increased mortality rates (Bunce et al. 1979). Moreover, a needle loss due to a cold winter can make a tree more susceptible to fungal attacks (Kullman 2007). Snow cover has a fundamental importance for many organisms living near the ground and can also protect the roots of trees from freezing temperatures (Sutinen et al. 1996). The amount of snow and its distribution can also affect the growing season in a profound way since certain areas in coldward positions or where the snow has accumulated can melt very late. Although organisms living in areas with a late snowmelt will have the disadvantages of shorter growing season, they might also experience a lower risk of detrimental spring frosts (Boggs and Inouye 2012). One example of the assumption that different species or varieties could be regulated by the same climatic conditions is maps describing different hardiness zones for crops and garden plants (e.g., McKenney et al. 2006). For example, these zones in northern USA are based on the spatial variation in extreme minimum temperatures (McKenney et al. 2006). However, this is not a general pattern for trees in Europe. A recent study showed that the distributions of several broadleaved trees are limited by low temperatures in the spring instead (Kollas et al. 2014). Toward the warmward (i.e., toward the warm end of a temperature gradient) end of ranges, other climate-related variables, such as a combination of high temperatures and drought killing individuals or increasing the susceptibility to insect attacks, have been suggested to limit species ranges (Hampe and Petit 2005; Gaylord et al. 2013). A common theme for these kinds of studies is the proposition that extreme climatic events (e.g., very cold events or droughts) often are important in regulating ranges (Allen and Breshears 1998; Niu et al. 2014). Mechanisms suggested to regulate range limits through effects on fecundity and recruitment have mostly focused on the conditions during the warm season. A classic example is lime *Tilia cordata*, which has been shown to be limited toward colder areas by that the pollen tube cannot grow under 15 °C (Pigott and Huntley 1981). Hence, no viable seeds are produced and the existence of adult trees in area with a temperature lower than 15 °C during the fertilization period indicates previous warmer climate.

In many cases, it might not be a specific threshold in temperature that is necessary, but instead an enough long warm period. A commonly used metric to capture the fact that an organism needs a certain amount of energy to complete development before overwintering is growing degree-days (GDD) (Bonhomme 2000). The rationale is that a shorter but warmer period could be as favorable as a longer but somewhat colder period. Many models are using GDD to predict the distribution of species (Austin 2007). Another related variable is the length of the frost-free season. This variable could for example be relevant in the case there is a threshold value when warmer temperatures are not favorable.

All species occur in a context of other species and the responses of a focal species to a changed climate will in most cases be determined by the combination of direct effects and changes in inter-specific interactions induced by climatic changes (Lenoir et al. 2010; Navarro-Cano et al. 2015). For example, competition is suggested to be more important as limiting factor under warm and moist than cold and dry conditions (Normand et al. 2009 and references therein; Pellissier et al. 2013). At least for plants, a warmer and moister environment will lead to denser vegetation and more intense competition (le Roux et al. 2014). Competition can constrain some vital rates so that a plant population might respond more positively to temperature when competition is reduced (Sletvold et al. 2013). Thus, there could still be a climatic component even if a species is influenced by interactions with other species (Choler et al. 2001; Hampe and Jump 2011; Meineri et al. 2012).

From the reasoning above, it is evident that different aspects of the climate might be limiting for different species through effects on different vital rates. In some cases, an effect of a specific climatic variable on a single vital rate might be crucial, but in most cases influences are likely to be much more complex. Species may be influenced by multiple climatic variables influencing different vital rates and these effects may sometimes be in opposed directions. For example, the population growth rate of a rare alpine herb was positively related to high spring temperature but negatively to high summer temperature (Nicole et al. 2011). Moreover, interactions between local factors and climate can lead to a spatial variation in relationships between a given climatic variable and population growth rates that is sometimes hard to predict (Lenoir et al. 2010). In the rare alpine herb mentioned above, population growth rate was more negatively affected by high summer temperatures on steep than on gentle slopes (Nicole et al. 2011). Similarly, the altitudinal optima for Californian mountain plants was determined by interactive effects of temperature and precipitation (Crimmins et al. 2011). It is thus possible that interactions with different local factors might determine not only the magnitude but also the direction of effects of climatic differences (in space or time). Such interaction effects can be both direct or occur through competitive or facilitative interactions with other organisms.

In this section, we have shown that many different climate-related variables could determine different species performances and thus their geographical distribution. Such regulations could act through constraints on a species physiology or be the outcome of the species’ interaction with other species. In order to assess the potential for a species to utilize a certain area (being it large or small), we thus need to know the environmental conditions determining performance. In the next section, we will examine how different climatic conditions are distributed across scales relevant for organisms and how such differences might influence the possibilities for survival in microrefugia when regional conditions change.

## Local versus regional climates

A key to understand where microrefugia are likely to be situated is knowledge of the relationship between local and regional climates (Dobrowski 2011). Over large spatial scales many climatic variables are correlated. For example, both maximum and minimum temperatures are higher in southern than in northern Europe. However, as scale decreases it is not necessarily true that these correlations remain. For example, the sites within a landscape having hottest summers are not necessarily the sites with warmest minimum temperatures (e.g., Fridley 2009; Ashcroft and Gollan 2012; Aalto et al. 2014). In contrast to many temperature variables, moisture variables could display everything from positive to negative correlations even across large scales.

To better understand local variation in climatic conditions, it is necessary to understand the climate-forcing factors (Dobrowski 2011; Ashcroft and Gollan 2012). A strong regulator of the local climate is elevation. Higher altitudes are associated with lower temperatures and the so-called “lapse rate” describes how temperature negatively correlates (5–7 °C/km) with elevation. However, many factors could moderate the local climate and sometimes even reverse the effect of altitude. One such example is cold-air drainage toward depressions in the landscape (Dobrowski 2011). This can lead to inversions where temperature instead increases with elevation. Variations in topography can modify the local climate also in many other respects. An important contrast among places in a topographically heterogeneous landscape is the variation in insolation at slopes with different angles to the sun (Huang et al. 2008). Other factors modifying the local climate include costal effects and distance to smaller water bodies (Vercauteren et al. 2013; Aalto et al. 2014), moisture, and vegetation (Ashcroft and Gollan 2012, 2013).

**Fig. 2:**
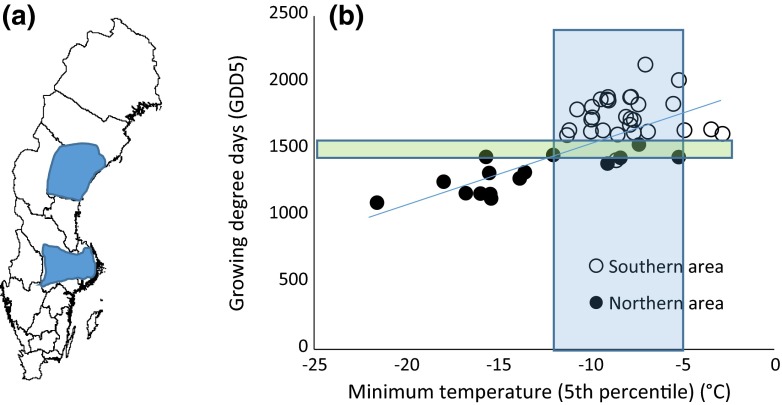
Variation in growing degree-days and minimum temperature across a latitudinal gradient in Sweden represented by two regions: in south (Mälardalen) and in north (central Norrland). Data are from one year from the SMHI stations. **a** The two regions in Sweden, **b** a trendline superimposed on the full data and *boxes* showing differences in the overlap between the two variables

Variation in many climatic variables can be large in a topographic heterogeneous landscape, which corresponds to considerable latitudinal distances in flat landscapes (Scherrer and Körner 2011; Lenoir et al. 2013). For example, a difference in mean soil temperature of 7.2 °C was recorded among microsites during the growing season within a 2-km^2^ area in the Alps (Scherrer and Körner 2011). However, it is likely that different climatic variables differ with regards to how well they can be “reproduced” in landscapes at different latitudes (or altitudes). In other words, different climatic variables will differ concerning how strongly local conditions are determined by the regional mean. To examine this in more detail, we used temperature data from 54 weather stations collected by the Swedish Meteorological and Hydrological Institute (SMHI). We extracted hourly temperature data from a twelve-month period (June 2010–May 2011) from weather stations in southern and northern Sweden (Fig. 2a). From these data, we calculated two variables: the 5 % percentile minimum temperature (hereafter minimum temperature) and growing degree-days (base 5 °C, hereafter GDD). It is obvious that over the whole gradient there is a clear correlation between these variables with higher coldest temperature in areas with high GDD (Fig. 2b). However, it is also clear that there is much more overlap in minimum temperatures between the regions than for GDD (Fig. 2b). According to our reasoning and these data, this would imply that species limited by GDD toward the north might have had more difficulty in finding localities with suitable conditions in microrefugia in northern Sweden during the last cooling period after the warm peak 5000 years ago, than species limited by minimum temperatures. The data used for this example are from weather stations, which are located at sites selected to well represent regional averages. If variation in micro-climate across a landscape had been based on random sites or stratified to capture extremes along e.g., topographic gradients, the variation in both variables would probably have been much larger in both of the regions. Thus, to assess the case illustrated in Fig. 2 in more detail would need additional data sampled at a much finer resolution and sampling covering a broad range of small-scale habitat variation.

In spite of recent attention to the question of how local climate is regulated by climate-forcing factors (Fridley 2009; Ashcroft and Gollan 2012), there is still much to learn. For example, to achieve a better understanding of the correlation of different factors between local and regional levels, there is a need for studies using micro-loggers stratified across gradients of impact from different climate-forcing factors simultaneously across small scales and across wide geographic areas/gradients. To predict when microrefugia are likely to play a role, we need to know both which variables that might be decoupled (i.e., has a different climate at the local versus the regional mean) and under which conditions they are decoupled (which might be different for different variables). Dobrowski (2011) suggested that concave environments such as valleys and depressions are most likely to have properties decoupling local from regional climates. Hilltops, however, will be exposed to winds that mix the air effectively so that the local climate will more closely follow the regional mean. Decoupling between the local and regional climate could also be due to several other factors, such as hydrology and vegetation (e.g., Ashcroft et al. 2012), and there is a need for studies of different combinations of regional climate and climate-forcing factors.

Also, the effects of local variation in moisture conditions need to be considered in studies of plants (Stephenson 1990). Many plants are limited by the availability of water, and soil moisture may vary markedly across topographic gradients of a few meters (le Roux et al. 2013). Also moisture conditions may thus potentially play a crucial role in determining which places will become microrefugia. For example, mist surrounding waterfalls could create conditions similar to an area with high precipitation. Soil moisture can also moderate temperatures (Aalto et al. 2014), and it might only be during really dry periods that other climate-forcing factors such as radiation have an effect on distribution of soil temperatures (Ashcroft and Gollan 2013). This suggest that ground-water fed areas might have low maximum temperatures and potentially can function as microrefugia for species sensitive to high summer temperatures in more equator-close localities. However, it has been suggested that regions with a generally moist climate might have fewer microrefugia, since the moisture buffers the gradients in extreme temperatures that are present in such landscapes compared to in drier landscapes (Ashcroft and Gollan 2013). Thus, not only temperature but also moisture (precipitation and cloudiness) will be important to determine the location of microrefugia.

The reasoning above considers spatial decoupling of the local and regional climate, i.e., a local site has a different climate than the regional mean climate. However, it is important to also consider whether the same areas that are spatially decoupled from the regional means also will be temporally decoupled, i.e., not all areas in a landscape will display a changed climate of the same magnitude as the regional change. It is likely that those climate-forcing factors that decouple some places from the regional mean also are involved in moderating climatic temporal trends (Ashcroft et al. 2009; Dobrowski 2011).

## Synthesis

Our brief review suggests that (1) distributions of different species are influenced by different climatic variables, and (2) some climatic factors can be strongly correlated between the local and the regional scale, while others are only weakly correlated. We argue that these two observations imply that while some species are likely to be able to benefit from microrefugia, other species will not. To benefit from microrefugia, species should be limited by those climatic factors that are decoupled from the regional climate. This implies that not all species can take similar advantage of microrefugia, since each species has a different climatic niche and only certain combinations of climatic conditions might be available. A microrefugia will not occur just because the site has a climate that is similar in some respects to the climate in the core range of the species (or like it was before the change if viewed in a temporal context) unless the species is able to persist or sustain a positive population growth rate in these sites. On the other hand, microrefugia do not require a climate that is completely analogous to that of the core range (or to the previous climate). Recently, Hannah et al. (2014) proposed the term “holdouts,” which are sites in which populations survive a changing climate during a period and then are likely to go extinct. This would be in contrast to microrefugia, which by definition are sites with populations that survive until the climate has returned so that they could later expand. Most of our points stressed here for microrefugia are valid also for holdouts, since the distinction between microrefugia and holdouts only could be judged from predictions of future changes or in retrospect.

The potential for microrefugia is expected to not only differ among species but also between changes toward warmer and colder climates. For example, a warmer climate might cause a decline of a warm-adapted species at its equatorward range edge due to competition while a decline during a cooling period might be due to physiological stress in its poleward range edge (cf. Pellissier et al. 2013). Thus the same species could have a different capacity of utilizing microrefugia in cases of a warming or a cooling of the climate. For species in which it is possible to investigate both the cold and warm-ward limits, it would be valuable to compare the distributional edge zones to the different directions. Perhaps the species will have many scattered localities indicating the potential for microrefugia toward one range edge but less toward the other (cf. Fig. 1b, c).

The proposition that there are one or a few non-interacting climatic variables limiting species distributions, and that spatial variation in these variables determines the locations of microrefugia, is likely to constitute a simplification, but one that is conceptually valuable. In reality, it is important to be aware of the difficulties associated with teasing apart the effects of different climatic factors on a species geographical limit (Gaston 2009). Moreover, there could be differences between the response of different vital rates to the same climatic change (Doak and Morris 2010), or contrasting responses to different aspects of the climate (e.g., spring vs. summer temperature Nicole et al. 2011). It is also important to realize that what seems to be a limiting factor for a species in one region (or during one time in history) not necessarily will be limiting in another region (time), e.g., because it fills different parts of its’ fundamental niche in different regions (cf. Colwell and Rangel 2009; Guisan et al. 2014).

Many researchers have proposed that a rough topography and the occurrence of microrefugia will enhance the resilience of species to climate change (Loarie et al. 2009; Ashcroft 2010; Keppel et al. 2012). We agree that this indeed could be the case, but would at the same time like to focus the attention to that there will be differences among species in how likely this would be. Improving our knowledge about both how vital rates of populations are linked to climate and how climate-forcing factors shape the correlation between local and regional climate across space and time is key to understand and predict the role of microrefugia in a changing climate. The fact that species differ in their climatic niches is widely recognized (e.g., Woodward 1987; Guisan et al. 2014) and differences between the local and the regional climate have recently gained much attention (Bennie et al. 2008; Fridley 2009; Ashcroft and Gollan 2012). Yet, in our opinion we need a more refined knowledge in both these fields. For example, we need to more explicitly consider the factors governing differences in correlations between local and regional conditions for specific climatic variables. Moreover, to evaluate the potential for microrefugia we need to study them simultaneously.
